# Genetic Heterogeneity of Oesophageal Cancer in High-Incidence Areas of Southern and Northern China

**DOI:** 10.1371/journal.pone.0009668

**Published:** 2010-03-15

**Authors:** Zhang Guohong, Su Min, Wang DuenMei, Hu Songnian, Liu Min, Li Jinsong, Lin Hongbin, Zhang Feng, Tian Dongping, Yang Heling, Liu Zhicai, Lian Shiyong, Guo Quansheng, Li Xiaoyun, Gao Yuxia

**Affiliations:** 1 Department of Pathology, Shantou University Medical College, Shantou, Guangdong Province, China; 2 Beijing Institute of Genomics, Chinese Academy of Sciences, Beijing, China; 3 Health Bureau of Nanao Island, Nanao, Guangdong Province, China; 4 Linzhou Tumor Hospital, Linzhou, Henan Province, China; The University of Hong Kong, Hong Kong

## Abstract

**Background and Objective:**

Oesophageal cancer is one of the most common and deadliest cancers worldwide. Our previous population-based study reported a high prevalence of oesophageal cancer in Chaoshan, Guangdong Province, China. Ancestors of the Chaoshan population migrated from the Taihang Mountain region of north-central China, which is another high-incidence area for oesophageal cancer. The purpose of the present study was to obtain evidence of inherited susceptibility to oesophageal cancer in the Chaoshan population, with reference to the Taihang Mountain population, with the eventual goal of molecular identification of the disease genes.

**Methods:**

We conducted familial correlation, commingling, and complex segregation analyses of 224 families from the Chaoshan population and 403 families from the Taihang population using the FPMM program of S.A.G.E. version 5.3.0. A second analysis focused on specific families having large numbers of affected individuals or early onset of the disease.

**Results:**

For the general population, moderate sib-sib correlation was noticed for esophageal cancer. Additionally, brother-brother correlation was even higher. Commingling analyses indicated that a three-component distribution model best accounts for the variation in age of onset of oesophageal cancer, and that a multifactorial model provides the best fit to the general population data. An autosomal dominant mode and a dominant or recessive major gene with polygenic inheritance were found to be the best models of inherited susceptibility to oesophageal cancer in some large families.

**Conclusions:**

The current results provide evidence for inherited susceptibility to oesophageal cancer in certain high-risk groups in China, and support efforts to identify the susceptibility genes.

## Introduction

The incidence of oesophageal cancer varies by more than 300-fold worldwide, with the highest rates recorded in certain areas of China and central Asia.[1], [2], [3] For most Chinese populations, the vast majority of oesophageal cancers are squamous cell carcinomas.[1], [4], [5] In north-central China, the high-incidence areas are mainly located along the northern borders of three provinces—Hebei, Henan, and Shanxi—abutting the southern flank of the Taihang Mountains. The mortality rate can be as high as 1100/100,000, as occurs in Linxian, Henan Province, and in Yangcheng, Shanxi Province.[6], [7], [8] Over the last two decades, Wu and his colleagues [9] have sought epidemiological evidence of genetic susceptibility in the development of oesophageal cancer as well as effective ways of screening for individuals who are highly susceptible to the disease. Familial aggregation of oesophageal cancer was found in this high-incidence area.[10] Moreover, the results of segregation studies by Wu et al.[7], [11] on 221 high-risk nuclear families from Linxian, all with offspring ≥40 years old, and 225 high-risk families collected from Yangquan City suggested an autosomal recessive mode of inheritance of oesophageal cancer in these two high-risk locales.

In southern China, in the Chaoshan littoral region two-thousand kilometers from the Taihang Mountains, there exists another high-incidence area for oesophageal cancer, notably Nanao Island.[4] According to our previous study, the age-standardised incidence rates of oesophageal cancer in males and females were 72-150/100,000 and 26-64/100,000, respectively, from 1995 to 2004 in Nanao Island. Oesophageal cancer and cardiac cancer were the most prevalent malignancies, comprising 52% of the total malignant tumour cases, and with oesophageal cancer showing an upward trend.[12]

Despite the geographic separation, could there be a common aetiology for oesophageal cancer in those two areas? Historical records, supported by recent genetic data in the form of polymorphisms of both the Y chromosome and mtDNA, indicate that the ancestors of the Chaoshan people migrated from the Taihang Mountain region. Analysis of mtDNA haplogroups showed that a shared maternal genetic background is associated with the high-risk populations in the two areas. [13]

Epidemiological studies have shown that certain environmental risk factors are also associated with oesophageal cancer in the Chaoshan area, including fermented fish sauce, dietary habits, alcohol consumption, tobacco smoking, and drinking of Kongfu tea.[6], [14], [15], [16], [17], [18], [19], [20], [21], [22] Nonetheless, familial clustering of oesophageal cancer has been documented in the Chaoshan high-incidence population, providing an important clue to genetic aetiology.[23] Moreover, the existence of two high-risk populations—Chaoshan and Taihang—in two obviously different environments, but which are related through common ancestors, indicates that genetic susceptibility may play an important role in the risk of developing oesophageal cancer. Therefore, it is essential to explore whether genetic factors are indeed involved in the aetiology of oesophageal cancer in the Chaoshan high-incidence area.

The aim of the present study was to obtain evidence of a specific model of inherited susceptibility to oesophageal cancer in the Chaoshan and Taihang Mountain high-risk populations that would support further studies to localise susceptibility genes in familial oesophageal cancer. A total of 224 population-based pedigrees from the Chaoshan area and 403 hospital-based pedigrees from the Taihang Mountain area were collected and studied for familial correlation, commingling, and complex segregation using the program Statistical Analysis for Genetic Epidemiology (S.A.G.E.).

## Materials and Methods

### Study subjects and data collection

Subjects from two populations were studied: population-based subjects of Chaoshan in southern China, and hospital-based subjects of the Taihang Mountain region in northern China. The Chaoshan area, with a population of approximately 10 million, is a littoral area in the eastern part of Guangdong Province. Its major cities are Shantou, Chaozhou, and Jieyang. Directly east is Fujian Province and to the south is the South China Sea. Chaoshan residents comprise a relatively isolated population and have kept the old Chinese language (Chaoshan dialect) and traditional customs. Nanao Island, with a population of approximately 70,000, is a county attached to Shantou, opposite Taiwan. The residents of Nanao Island are participants in a long-term study of oesophageal cancer, and a cancer registry for Nanao Island has been operating in cooperation with the Department of Pathology, Shantou University Medical College and the Health Bureau of Nanao Island since 1995. The system is a large population-based network covering village clinics as well as town and county hospitals. Recorded information includes patient demographics and native origin, age of onset, X-ray and pathologic confirmation of diagnosis, and stage of the disease for each case of oesophageal and cardiac cancer. Guided by the records and after obtaining informed consent, we interviewed all new subjects at their homes accompanied by staff of the Board of Health of Nanao Island. A structured questionnaire was administered to the patients, who were asked to provide verbal answers. We recorded the information, including lifestyle habits (e.g., tobacco smoking and alcohol drinking) and diet, as well as the family history of four successive generations. Whenever a proband was unsure of an answer, we interviewed additional family members or older neighbors to ensure the accuracy of the information. Because difficulty in swallowing is an important clinical symptom, it was used in the diagnosis of the disease; before the 1980s, when poor medical conditions prevailed, the older oesophageal cancer patients who were diagnosed by village doctors or suspected of having died of EC had been ascertained by this method. From this population, a total of 224 oesophageal cancer and cardiac cancer pedigrees were enrolled from registry data collected in 2003 - 2005.

The Taihang Mountain area has the highest incidence of oesophageal cancer in northern China. For this region we chose a cross-sectional study of a hospital-based population. We investigated the medical records of all patients with oesophageal cancer who presented at four hospitals—Linzhou (Linxian) Tumour Hospital, Linzhou People Hospital, Anyang Centre Hospital, and Anyang Tumour Hospital—during June to August 2005. After receiving consent from the patients and their physicians, we interviewed the patients. Families were ascertained through single probands, with the most recent affected family member being selected as the proband. Familial occurrence of oesophageal cancer was investigated in face-to-face interviews. Date at diagnosis was recorded for the proband and for all affected relatives. A total of 7379 individuals in 403 families ascertained through probands were enrolled in the study.

To explore the heterogeneity of age at onset we used the following criteria for selection of large pedigrees: oesophageal cancer patients in three successive generations, and age of onset progressively decreased from generation to generation. Finally, we obtained two early-onset families from 224 pedigrees in Chaoshan, and four early-onset families and a single extended pedigree (six generations deep) with 32 of 293 individuals affected with oesophageal cancer in the Taihang Mountains.

Informed-consent documents were signed by each participant before entering the project. This study was approved by the ethical review committee of Shantou University.

### Familial correlation and statistical analysis

Familial correlation analysis was performed by using FCOR; version 5.3.0 of S.A.G.E. FCOR was used to estimate the correlations in trait values between pairs of relatives. Here, oesophageal cancer status was fixed as a trait; pair correlations were estimated and concentrated on the following relative types: parents and offspring, siblings, avuncular, and cousins. In addition, Chi-square statistics and P-values were calculated to test the homogeneity of correlation among the subtypes within each main type.

### Commingling analysis

The covariance structure was analysed by using the Commingling segregation program, version 5.3.0 of S.A.G.E.. In setting parameters, one-, two-, and three-component distributions were estimated, separately. For each distribution, no transmission was specified. The other parameters for each component mean were estimated by the maximum likelihood method, in which all numerical methods start from a given set of initial values of the unknown parameters, and then begin traversing the likelihood surface until a boundary is met, a local maximum has been found, or numerical difficulties are encountered.

### Complex segregation analysis

We conducted a complex segregation analysis on the families to model genetic susceptibility to and age at onset of oesophageal cancer by using the class finite polygenic mixed model (FPMM), which is the only option currently available for binary traits with variable age of onset under a Linux 3.0 operating system, version 5.3.0 of S.A.G.E.

This model leads to a likelihood that can be calculated using efficient algorithms developed for oligogenic models. The profiles for FPMM were closest to the profiles for the usual mixed model with exact calculations.[24], [25], [26] The basic theory of complex segregation analysis is that a subset of individuals in the population is assumed to be susceptible to oesophageal cancer and therefore to have an age of onset of oesophageal cancer, providing they live long enough, whereas the distribution of age of onset might depend on the possible segregating genes. If Mendelian transmission exists, it is assumed to be through a single autosomal locus with two alleles, A and B, A being the hypothesized disease allele. The frequencies of allele A and B are denoted q_A_ and (1- q_A_), respectively. The segregation of a possible major locus is allowed for by letting one or more parameters depend on an unobserved (latent) qualitative factor u  =  AA, AB or BB. We call u an individual's type. In this context, type is best defined in terms of the expected distribution of an individual's offspring. Thus we use the term type to allow for many kinds of discrete transmission, whether Mendelian or not. The distribution of types in the population is assumed to be in Hardy-Weinberg equilibrium. Individuals of each type are assumed to transmit allele A to their offspring with transmission probabilities τ_AA_, τ_AB_, and τ_BB_, respectively. Letting β be a baseline parameter and α the age coefficient.

In this study, age, oesophageal cancer status, and sex were included to improve model fitting; cancer status was considered to be primary binary, i.e., 0 for no oesophageal cancer and 1 for cancer, and sex-code was fitted as a covariate to a primary trait, i.e., 0 for male and 1 for female. Under FPMM of segregation analysis, four major Mendelian gene models (dominant, recessive, codominant, descending), polygenetic models (pure polygenetic, major gene and polygenetic) and three non-genetic models (no major type, pure environmental, and general) are implemented on the data to estimate which model is most consistent with the observed data. **There nine hypothesized models are following:**

1. Mendelian models: Mendelian transmission of a major gene is assumed in this model (τ_AA_ = 1, τ_AB_ = 0.5, τ_BB_ = 0). In the *dominant* model, genotype AA is equivalent to genotype AB, as reflected by the baseline parameters β_AA_ = β_AB_. In the *recessive* model, genotype BB is the same as the genotype AB, as reflected by the baseline parameters β_BB_ = β_AB_. In the *codominant* model, genotype AB is intermediate to genotypes AA and BB, as reflected by the baseline parameters β_AB_ = 1/2(β_AA_+β_BB_). In the *descending* model, genotype β_AA_≥β_AB_≥β_BB_.
2. Nongenetic models: an environmental model is also fit that includes a major type effect that is not transmitted from parent to offspring: A nontransmitted environmental effect model, in which each of the transmission probabilities is taken to be equal to the frequency of allele A, i.e., τAA  =  τAB  =  τBB  =  qA. In the other environmental model, which allows for possible heterogeneity of exposure levels between generations, the transmission probabilities are taken to be equal, i.e., τAA  =  τAB  =  τBB; No major type model, no transmission and no major gene and environment-type effects; General (multifactorial) model, a full model with arbitrary transmission probabilities, in which all parameters were unrestricted and allowed to fit the data.
3. Polygenetic or major gene and polygenetic models: option of the age of onset or susceptibility has a polygenic component specified under the FPMM block. In the program, the type frequencies, baseline parameters (β), and transmission probabilities were estimated. The model with the lowest Akaike's information criteria (AIC) value was considered to be the best model, supported by likelihood ratio tests.

To explore for ascertainment bias and a single or a multipoint ascertainment, the model-free likelihood of each pedigree was conditioned on the proband by age at onset and estimated. The Commingling segregation analysis program can be used to fit mixtures of two or three normal distributions, simultaneously applying a power transformation to the data and also allowing for both ascertainment and residual familial correlations.[27]

To compare with the results of the former study under a class D model, we generated the following covariates representing residual familial effects as described by Carter et al.[11] Briefly, these are F1 (affected father effect), M1 (affected mother effect), S1 (number of affected older sibs); F1, M1, and S1 were coded 1 if father, mother or sibs were affected, respectively, and 0 if unaffected or missing.

## Results

In Chaoshan, of 7224 individuals in 224 pedigrees, 374 (5.18%) (including probands) had oesophageal cancer (table 1). In Taihang, of 7379 individuals in 402 pedigrees, 738 (10.0%) had oesophageal cancer. The pedigrees in Chaoshan, based on population data, tended to be large and multigenerational (>3 generations), whereas those ascertained from hospital-based data in Taihang included many nuclear families of two or three generations only. These differences in pedigree structure may account for the apparent twofold difference in the rate of oesophageal cancer between the two study groups.

**Table 1:** The descriptive statistic of pedigrees.

	Populations		Large pedigrees		Extended pedigree
	Chaoshan	Taihang	Chaoshan	Taihang	
**individuals**	7224	7379	62	158	293
affected	374	738	8	25	32
Male	268(71.66%)	458(62.06%)	5(62.5%)	16(64.0%)	17(53.13%)
Female	106(28.34%)	280(37.94%)	3(37.5%)	9(36.0%)	15(46.87%)
Mean of age-of-onset	60.75±11.32	61.65±10.11	49.25±8.59	59.78±11.05	51.68±9.90
Male	59.94±11.35	61.08±10.05	47.00±7.51	59.5±12.76	51.94±10.29
Female	62.78±11.03	62.53±10.18	53.00±10.58	60.28±7.61	51.40±9.79
**pedigrees**	224	403	2	4	1
One affected	134	228			
Two affected	72	100			
Three affected	12	32			
More three affected	6	43	2	4	1

Descriptive characteristics of the pedigrees are presented in table 1. More males than females were affected in both populations. The proportion of pedigrees with multiple (≥2) affected members was similar between the two populations (40.18% in Chaoshan, 43.42% in Taihang, *P*>0.05). The mean age of onset (at diagnosis) of oesophageal cancer in Taihang (61.65 years) was slightly higher than in Chaoshan (60.75 years, *P*>0.05).

### Familial correlations

Familial correlation analysis was performed by using FCOR (S.A.G.E. version 5.3) to estimate correlations in trait values between pairs of relatives as described in the Materials and methods. We found that all correlation coefficient values for the Chaoshan population relative pairs were similar to those for the Taihang population (table 2). Overall, higher sibling–sibling than parent–offspring correlations were found, suggesting familial clustering of the genes, with each generation possibly having a different environment. The range of sibling correlations was 0.079 to 0.149 in Chaoshan and 0.102 to 0.171 in Taihang, with the **brother–brother** correlation coefficient being relatively high as compared with the other sib–sib correlations. These results indicate the involvement of genetic influences in the aetiology of oesophageal cancer in the general population.

**Table 2:** Familial correlation coefficients and standard error for EC among the various familial pairs.

	Populations				Large pedigrees				Extended pedigree	
	Chaoshan		Taihang		Chaoshan		Taihang			
	Pairs	Correlation Coefficient	Pairs	Correlation Coefficient	Pairs	Correlation Coefficient	Pairs	Correlation Coefficient	Pairs	Correlation Coefficient
Parent-offspring	10234	0.022 (0.013)	10876	0.039 (0.013)	90	0.171 (0.103)	176	0.191 (0.067)	218	0.195 (0.044)
Father-son	2812	0.026 (0.015)	2507	0.062 (0.027)	27	−0.073 (0.249)	48	0.208 (0.076)	118	0.263 (0.079)
Father-daughter	2305	0.029 (0.014)	2931	0.042 (0.029)	18	0.080 (0.023)	40	0.235 (0.078)	101	0.253 (0.094)
Mother-son	2812	0.048 (0.028)	2507	0.061 (0.026)	27	0.606 (0.148)	48	0.160 (0.053)	118	0.101 (0.074)
Mother-daughter	2305	0.011 (0.008)	2931	0.046 (0.029)	18	0.125 (0.071)	40	0.148 (0.093)	101	0.178 (0.111)
Sib-sib	6365	0.102 (0.029)	8965	0.140 (0.020)	67	0.261 (0.178)	108	0.249 (0.108)	328	0.298 (0.127)
Brother-brother	1854	0.149 (0.033)	2033	0.171 (0.032)	23	0.488 (0.237)	38	0.338 (0.102)	80	0.159 (0.198)
Brother-sister	3168	0.107 (0.025)	4544	0.136 (0.024)	35	0.217 (0.179)	58	0.237 (0.100)	186	0.316 (0.098)
Sister-sister	1343	0.079 (0.038)	2388	0.102 (0.026)	9	−0.200 (0.260)	22	0.235(0.112)	62	0.416 (0.233)
Avuncular	15531	0.006 (0.002)	12487	0.005 (0.002)	174	*******	156	0.074 (0.016)	1086	0.021 (******)
Cousin	18247	0.007 (0.002)	12092	0.008 (0.003)	213	*******	96	0.084 (0.024)	1635	0.057 (0.087)

*******: Value is not estimable.

For the large families, all correlation coefficient values were higher than those for the general population. The highest correlation coefficients were for the brother–brother (0.488) and sister–sister (0.416) pairs, indicating a strong genetic effect on susceptibility to oesophageal cancer in the large families of both the Chanshan and Taihang areas (table 2).

### Commingling analysis

**Figure 1:**
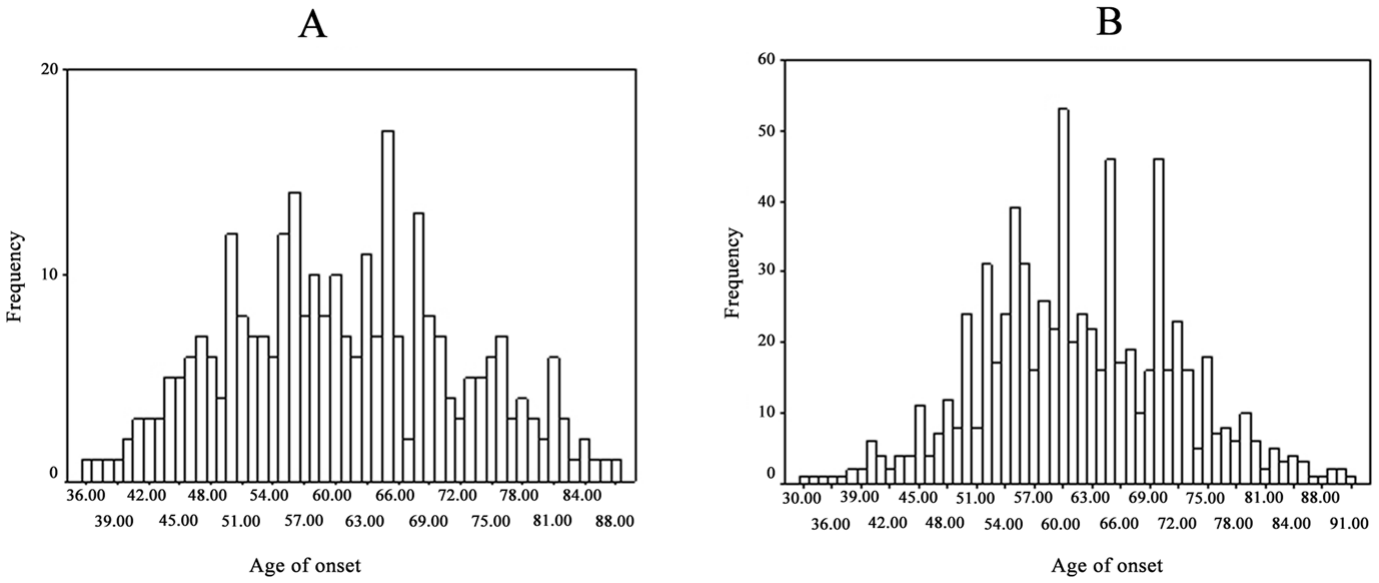
The distribution of age of onset in the two general populations. Panel A: the general population of Chaoshan, Panel B: the general population of Taihang. The histograms show there is a mixture of distributions in the two populations.

Commingling analysis was carried out using the Commingling segregation program of S.A.G.E. (version 5.3.0) to assess whether the distribution of the mean age at onset of oesophageal cancer in the families could be better explained as a mixture of two or more distributions rather than a single distribution, and to determine the number of means to fit in the segregation analysis (see below). The results of commingling analysis are presented in fig. 1.

A mixture of two distributions fits significantly better than a single distribution (Chaoshan: χ^2^ = 30.09, *P*<0.001, Taihang: χ^2^ = 48.15, *P*<0.001). A mixture of three distributions with three arbitrary means has the largest likelihood and fits the data slightly better than the two-distribution model in Chaoshan (general population χ^2^ = 4.1, *P* = 0.04288) and obviously better in Taihang (χ^2^ = 19.54, *P*<0.001). Thus, the commingling distributions of the two populations are better explained by a mixed distribution than a single one and are compatible with a possible major gene effect (fig. 1).

### Segregation analysis

#### Segregation in the general population

To determine a possible mode of inheritance we fitted nine models, including four Mendelian models, a polygenic model, a polygenic plus Mendelian model, an environmental model, no major type, and a full model with arbitrary transmission probabilities (general) as described in Materials and methods. The estimated parameters and the corresponding models used for complex segregation analyses of oesophageal cancer in both general populations were the same, and the results are presented in tables 3 and 4. For both populations, all *P*-values were less than 0.05, and the AIC values were lowest in the general models (3870.98 in Chaoshan and 7562.54 in Taihang Mountain). Excluding the general model, we found that the codominant model (4036.51) in the Chaoshan population and the pure polygenetic model (7896.56) in the Taihang population had the lowest AIC values. The results from both general populations support the multifactorial model as the best model.

**Table 3:** Segregation analysis of oesophageal cancer in the Chaoshan general population with type influences age of onset and sex-dependence.

Parameter^a^	Dominant	Recessive	Codominant	Descending	Polygenic	No major type	Environmental	General
q_A_	0.450	0.817	0.533	0.450	0.531	-	0.520	0.057
τ_AA_	1.0	1.0	1.0	1.0	-	-	0.520	0.924
τ_AB_	0.5	0.5	0.5	0.5	-	-	0.520	0.312
τ_BB_	0	0	0	0	-	-	0.520	0.849
β_AAM_	−12.626	−12.361	−13.005	−12.623	−17.892	−10.133	−13.415	−19.045
β_ABM_	−12.626	−16.006	−18.563	−16.612	−23.696	−10.133	−17.603	−12.321
β_BBM_	−16.615	−16.006	−15.896	−16.612	−27.988	−10.133	−20.517	−15.985
β_AAF_	−14.514	−14.130	−15.064	−14.513	−20.679	−11.460	−15.548	−20.467
β_ABF_	−14.514	−17.776	−20.623	−18.501	−26.482	−11.460	−19.736	−13.742
β_BBF_	−18.504	−17.776	−17.955	−18.501	−30.774	−11.460	−22.650	−17.407
α	0.211	0.205	0.223	0.212	0.307	0.137	0.230	0.202
γ	0.259	0.151	0.515	0.260	0.914	0.388	0.704	2.364
χ^2^	179.68	186.66	171.53	179.68	174.64	228.01	179.02	
Df	5	4	3	3	3	7	3	
*P* value	6.26E-37	2.77E-39	5.95E-37	1.03E-38	1.27E-37	1.31E-45	1.44E-38	
-2lnL	4028.66	4035.64	4020.51	4028.66	4023.62	4076.99	4028.00	3848.98
AIC	4040.66	4049.64	4036.51	4044.66	4039.62	4084.99	4044.00	3870.98

-: Parameter at this value is not estimated.

a: See Materials and Methods for definitions of the parameters.

**Table 4:** Segregation analysis of oesophageal cancer in the Taihang general population with type influences age of onset and sex-dependence.

Parameter^a^	Dominant	Recessive	Codominant	Descending	Polygenic	No major type	Environmental	General
q_A_	0.355	0.644	0.481	0.352	0.486	-	0.486	0.859
τ_AA_	1.0	1.0	1.0	1.0	-	-	0.486	0.49
τ_AB_	0.5	0.5	0.5	0.5	-	-	0.486	0.48
τ_BB_	0	0	0	0	-	-	0.486	0
β_AAM_	−12.729	−12.729	−13.194	−12.729	−14.827	−10.770	−14.827	−12.980
β_ABM_	−12.729	−15.785	−17.472	−15.785	−18.463	−10.770	−18.463	−16.017
β_BBM_	−15.785	−15.785	−14.774	−15.785	−22.293	−10.770	−22.294	−22.071
β_AAF_	−13.540	−13.540	−14.109	−13.540	−15.822	−11.424	−15.822	−13.565
β_ABF_	−13.540	−16.596	−18.387	−16.596	−19.458	−11.424	−19.458	−16.602
β_BBF_	−16.596	−16.596	−15.689	−16.596	−23.288	−11.424	−23.289	−22.656
α	0.210	0.21	0.224	0.21	0.259	0.155	0.259	0.219
γ	0^b^	0 b	0.100	0 b	0.216	0.018	0.216	0.859
χ^2^	364.85	364.88	349.79	364.82	340.02	405.04	340.02	
df	3	3	2	2	3	5	2	
*P* value	9.07E-79	8.94E-79	1.11E-76	6.03E-80	2.16E-73	2.43E-85	1.46E-74	
-2lnL	7907.39	7907.42	7892.33	7907.36	7882.56	7947.58	7882.56	7542.54
AIC	7921.39	7921.42	7908.33	7923.36	7896.56	7957.58	7898.56	7562.54

-: Parameter at this value is not estimated.

a: See Materials and Methods for definitions of the parameters.

b: Parameter estimate went to bound.

#### Segregation in large and extended pedigrees

In the large pedigrees from both areas, the Mendelian transmission models had the lowest AIC scores; the Chi-square and corresponding *P*-values are presented in tables 5 and 6. These results suggest that a major gene model underlies the aetiology of oesophageal cancer in these families. For the two large pedigree of Chaoshan, the models with the smallest AIC values were: recessive, dominant, and dominant with a polygenic mode of inheritance (table 5). For the four large pedigrees of Taihang, the recessive with a polygenic mode gave the smallest AIC value and is considered the best-fit model for those large families (table 6). In the extended pedigrees from Taihang Mountain, the Mendelian dominant models (table 7).

**Table 5:** Segregation analysis of oesophageal cancer in two large pedigrees of Chaoshan.

Parameter^a^	Dominant	Recessive	Codominant	Descending	Polygenic	Dominant + polygenic	No major type	Environmental	General
q_A_	0.121	0.121	0.128	0.129	0.399	0.192	-	0.600	0.916
τ_AA_	1	1	1	1	-	1	-	0.600	0.32
τ_AB_	0.5	0.5	0.5	0.5	-	0.5	-	0.600	0.55
τ_BB_	1	0	0	0	-	0	-	0.600	1.00
β_AA_	−10.431	−10.463	−13.508	−11.678	−20.107	−56.012	−9.794	−20.102	−0.269
β_AB_	−10.431	−52.790	−10.889	−92.812	−24.879	−56.012	−9.794	−24.875	−24.866
β_BB_	−50.639	−52.790	−54.497	−92.812	−32.353	−62.334	−9.794	−32.348	−32.955
α	0.198	0.199	0.209	0.216	0.492	1^b^	0.179	0.491	0.485
γ	1^b^	1^b^	1^b^	1^b^	0 b	0 b	0.140	0 b	0.385
χ^2^	4.869	4.911	3.962	4.889	4.800	2.258	9.392	5.941	
df	3	3	2	2	3	3	3	2	
*P* value	0.181	0.178	0.138	0.086	0.187	0.52	0.024	0.050	
-2lnL	80.101	80.143	79.194	80.121	81.173	77.49	84.624	81.173	75.232
AIC	92.101	92.143	93.194	94.121	93.173	89.49	96.624	95.173	93.232

-: Parameter at this value is not estimated.

a: See Materials and Methods for definitions of the parameters.

b: Parameter estimate went to bound.

**Table 6:** Segregation analysis of oesophageal cancer in four large pedigrees of Taihang.

Parameter^a^	Dominant	Recessive	Codominant	Descending	Polygenic	Recessive +polygenic	No major type	Environmental	General
qA	0.322	0.303	0.054	0.304	0.164	0.372	-	0.843	0.591
τAA	1	1	1	1	-	1	-	0.843	1.00
τAB	0.5	0.5	0.5	0.5	-	0.5	-	0.843	0.755
τBB	1	0	0	0	-	0	-	0.843	0
βAA	−7.282	−5.686	−0.977	−5.686	−6.870	−9.761	−8.437	−7.104	−9.160
βAB	−7.282	−12.542	−6.577	−12.542	−17.270	−18.551	−8.437	−16.863	−28.625
βBB	−15.915	−12.542	−14.130	−12.542	−25.213	−18.551	−8.437	−25.218	−21.306
α	0.214	0.159	0.189	0.159	0.282	0.251	0.108	0.287	0.308
γ	1^b^	1^b^	1^b^	1^b^	1^b^	1^b^	1^b^	1^b^	1b
χ^2^	13.336	15.595	17.983	15.595	5.859	1.814	20.191	8.213	
df	2	2	2	1	1	2	4	1	
P value	0.001	0.0004	0.0001	7.85E-05	0.0154	0.4037	0.0004	0.0041	
-2lnL	203.822	206.081	208.469	206.081	196.345	192.3	210.677	198.699	190.486
AIC	221.822	224.081	226.469	226.081	216.345	210.3	224.677	218.699	212.486

-: Parameter at this value is not estimated.

a: See Materials and Methods for definitions of the parameters.

b: Parameter estimate went to bound.

**Table 7:** Segregation analysis of EC in an extended pedigree of Taihang.

Parameter^a^	Dominant	Recessive	Codominant	Descending	Polygenic	No Major type	Environmental	General
q_A_	0.34	0.36	0.272	0.363	0.545	-	0.540	0.72
τ_AA_	1.0	1.0	1.0	1.0	-	-	0.540	0.84
τ_AB_	0.5	0.5	0.5	0.5	-	-	0.540	0.51
τ_BB_	0	0	0	0	-	-	0.540	0.17
β_AA_	−9.11	−9.94	−9.83	−9.460	−22.157	−9.48	−18.540	−18.06
β_AB_	−9.11	−35.04	−12.74	−54.531	−30.343	−9.48	−24.527	−24.01
β_BB_	−33.76	−35.04	−48.79	−55.911	−36.176	−9.48	−29.698	−542.31
α	0.157	0.163	0.204	0.163	0.488	0.151	0.405	0.386
γ	1^b^	1^b^	1^b^	1^b^	0.275	0.13	0.175	1^b^
χ^2^	0.541	3.482	3.525	3.482	4.9	14.409	11.224	
Df	4	4	3	3	3	6	5	
*P* value	0.969	0.481	0.317	0.323	0.179	0.025	0.047	
-2lnL	330.628	333.569	333.612	333.569	334.987	344.496	341.311	330.087
AIC	342.628	345.569	347.612	347.569	348.987	352.496	351.311	350.087

-: Parameter at this value is not estimated.

a: See Materials and Methods for definitions of the parameters.

b: Parameter estimate went to bound.

## Discussion

### A multifactorial mode of inheritance is the best model for susceptibility to oesophageal cancer in the two general populations

The results of the FCOR analysis confirmed that oesophageal cancer is moderately correlated amongst family members, and support the involvement of a genetic component for oesophageal cancer susceptibility in the two general populations. The moderate sister–sister correlation found in this study indicates that offspring had influential effects from their parents. Our results are consistent with those of previous studies showing familial correlation and aggregation of oesophageal cancer in families in the two populations.[10], [23]

The most satisfactory model from the commingling analysis for the two general populations consists of a mixture of three distributions, indicating that more than one component is needed to explain the distribution of oesophageal cancer. Our results are consistent with those of previous familial correlation and commingling studies, which indicated that a genetic risk factor(s) may be very important in the high-incidence areas of China.[9] The finding of multiple distributions is compatible with a major gene hypothesis; however, commingling may also arise through other causes. Thus, segregation analysis was used to determine whether these major effects segregated in families according to Mendelian expectations.

Development of advanced methods of segregation analysis and studying additional samples from other high-risk areas may be of help in better understanding the aetiology of oesophageal cancer.[7] In the Taihang Mountain area, a previous segregation analysis was performed using the REGTL program under a class D regressive model, and an autosomal recessive major gene was suggested[7]. However, until now, a similar formal segregation analysis had not been performed on the Chaoshan population. In the present study, we performed a complex segregation analysis using the SEGREG program under the FPMM model. Recent improvements to SEGREG give it significant advantages over the programs REGC, REGD, and REGTL of the previous S.A.G.E. versions; and FPMM, which is the only option currently available for binary traits with variable age of onset, is more effective than the class A regressive model for the data of esophageal disease as a main trait and age of onset as a censored trait. [28]

When we modelled age of onset, all Mendelian models, the pure polygene model, and the pure environmental model were rejected, whereas the general model was accepted at the population level in the two high-incidence areas. Although we did not find evidence supporting involvement of a major gene in the aetiology of oesophageal cancer in either of the populations, our results of multifactorial inheritance suggest that a variety of polygenes and environmental factors contribute to the disease. Furthermore, in the general population, the environmental model had the lowest AIC value next to the general model, suggesting there is an important role for environmental factors in the development of oesophageal cancer.

Our results are consistent with and support the opinion of Garavello et al.[7], [29], that a family history of cancer in combination with smoking and drinking increase the risk of oesophageal cancer. Additional genetic models need to be considered, including an interaction of susceptibility genes and environmental risk factors at the population level.

Segregation analysis is typically sensitive to ascertainment bias, and false assumptions on ascertainment could invalidate the estimates obtained through segregation analysis. An ascertainment correction was applied by conditioning the likelihood on the probands in the Taihang population.

### A major Mendelian component confers susceptibility to oesophageal cancer in large families

After analysing the two general populations, our second approach was to focus on families that have a history of oesophageal cancer, including families with large numbers of affected individuals or early age of onset. From Taihang we evaluated a kindred of 293 individuals with 32 affected members as well as four other large pedigrees; from Chaoshan we studied two large pedigrees whose average age of onset was 49.25 years.

The familial correlation and commingling analyses demonstrated that more than one component is needed to explain the distributions, with support for a genetic component. In particular, the high sib–sib correlations (r = 0.2610 and 0.2982) found in the large families indicates an important role for parental factors in influencing the offsprings' traits.

By complex segregation analysis of one large pedigree from Taihang and two large pedigrees from Chaoshan, both pure environmental and pure polygenic models were rejected; autosomal dominant and dominant with polygenic models were the best-fit patterns. For the other four pedigrees of Taihang, a recessive with polygenic model was the best model.

The results support the existence of a major susceptibility locus with Mendelian inheritance in some large families.

### Genetic heterogeneity of oesophageal cancer in the two high-incidence areas

In a study by Wu et al,[7] to explore heterogeneity in the age at onset of oesophageal cancer, the authors separated families into two groups, one with probands whose age at onset was <60 years and the other with probands whose age at onset was >60 years; they found no evidence of significant heterogeneity between these two subsets. Identification of subsets of families with different probable genetic aetiologies for oesophogeal cancer is taken as evidence of genetic heterogeneity. In this study, not only the age of the subjects but also the affected numbers of individuals, the generations with the affected individuals, and age variance were taken into account in chosing the subsets for analysis. Our data indicate a non-Mendelian mode of inheritance in the general population and an apparent dominant mode of inheritance in certain large families. These findings suggest that oesophageal cancer has significant genetic heterogeneity, specifically multi-factorial inheritance in the overall population and autosomal dominant inheritance in some subgroups of the population. Furthermore, results of previous studies suggested involvement of a major recessive gene in oesophageal cancer in Yangquan, Shanxi Province and Linxian, Henan Province.[7], [11] There is also evidence of a familial oesophageal cancer susceptibility gene region on chromosome 13q.[30], [31], [32], [33] Moreover, tumours from patients with a positive family history exhibit more frequent loss of heterozygosity (LOH) for this chromosome than do those from patients without a family history.[34]

As for other cancers, a dominant mode of inheritance of oesoaphageal cancer evident in a few very large families suggests involvement of common susceptibility genes. In other familial cases of oesophageal cancer and in sporadic cases, a polygenic/multifactorial aetiology can be postulated through sharing of alleles at many loci, each contributing to a small increase in cancer risk.[35] For example, only a subset of familial breast cancer is clearly hereditary, owing primarily to mutations in a single gene. Information about the genetic heterogeneity in oesophageal cancer is important to be able to identify the different subgroups and eventually reveal the disease loci.

### Further studies of genetic linkage and susceptibility gene location

Combined molecular genetic analysis and genetic epidemiology may reveal the underlying basis of the genetic predisposition to oesophageal cancer. Currently, a rare autosomal dominant disorder defined by a genetic abnormality on chromosome 17q25 is the only recognised familial syndrome that predisposes patients to squamous cell carcinoma of the oesophagus.[36] The present study has provided some modeling parameters of oesophageal cancer for further genetic linkage studies. Linkage and association studies aimed at localising the susceptibility genes involved in the development of oesophageal cancer in the Chinese high-risk populations are currently under way.
